# Risk factors for low knowledge and negative attitudes among caregivers of children with autism spectrum disorder in Iraq: a multi-centre cross-sectional study

**DOI:** 10.3389/fpsyt.2025.1568467

**Published:** 2025-08-04

**Authors:** Hiba Qahttan Khaleel Al-Juhaishi, Osamah Abbas Jaber, Faris Lami, Shatha Mohammed Jasim, Nahid Dehghan Nayeri, Mahdi Shafiee Sabet, Ghaith Al-Gburi

**Affiliations:** ^1^ Al-Subtain Academy for Autism and Neurodevelopmental Disorders, Karbala, Iraq; ^2^ Baghdad Teaching Hospital, Baghdad, Iraq; ^3^ Al-Subtain University of Medical Sciences, International Branch of Tehran University for Medical Sciences, Karbala, Iraq; ^4^ Department of Community Medicine, College of Medicine, University of Baghdad, Baghdad, Iraq; ^5^ Nursing and Midwifery Care Research Centre, School of Nursing and Midwifery, Tehran University of Medical Sciences, Tehran, Iran; ^6^ Department of Family Medicine, School of Medicine, Tehran University of Medical Sciences, Tehran, Iran; ^7^ School of Biosciences, University of Birmingham, Birmingham, United Kingdom

**Keywords:** autism, attitude, caregiver, knowledge, stigma, psychoeducation

## Abstract

**Introduction:**

Children with autism spectrum disorder (ASD) can experience delayed diagnosis and ineffective treatment due to low caregiver knowledge. Negative attitudes have also been linked to increased emotional problems and poor adaptive outcomes. Before educational interventions can address these issues, local knowledge and attitudes must be assessed, including the identification of high-risk groups that require prioritisation.

**Methods:**

Between February 17 and September 10, 2024, Al-Subtain Academy for Autism and Baghdad’s National Centre for Autism and Child Psychiatry conducted a cross-sectional study. Data was collected using a structured questionnaire developed based on the Autism Stigma and Knowledge Questionnaire (ASK-Q) and a review of previous studies. The questionnaire was pre-tested on 25 caregivers for clarity and reliability.

**Results:**

302 caregivers were included, all of whom were family members of the children. 57 caregivers (18.9%) had low knowledge of ASD, while only 24 (7.9%) had high knowledge. College-educated caregivers scored higher than those who were illiterate or with primary or secondary school education (p-values = 0.009, 0.002, and 0.007). Similarly, caregivers from low-income backgrounds had less knowledge than those from average and high-income backgrounds (p-value = 0.002 and 0.005). However, this difference was no longer apparent when controlling for the lack of tertiary education (B = 1.231, p-value = 0.119). 105 (34.8%) thought that a complete cure is possible, with higher rates among caregivers without tertiary education (43.0%, adjusted p-value = 0.048) or from low-income backgrounds (55.0%, adjusted p-value = 0.0002). In terms of attitudes, 44 (14.6%) believed that all children with ASD are aggressive, and 114 (37.7%) believed that they are deliberately negativistic and non-compliant. Being ashamed of the diagnosis was more common if the child had comorbid conditions. However, this difference was only significant before adjusting for multiple testing (adjusted p-value = 0.286).

**Conclusion:**

Educational programmes should be implemented to enhance knowledge and address treatment expectations, especially among caregivers with low income and lower education. Efforts should be focused on reducing negative attitudes to improve overall outcomes.

## Introduction

Children with autism spectrum disorder (ASD) may display a range of behaviours with different types and severities. As specified in the Diagnostic and Statistical Manual of Mental Disorders, Fifth Edition (DSM-5), these behaviours encompass difficulties in social reciprocity and both verbal and nonverbal communication, as well as repetitive and restrictive behaviours and interests (1). Although there are some epidemiological differences between genders (with males having higher rates) and slight variations across races, ASD can affect individuals of all genders and nationalities (2). A meta-analysis, utilising data up to 2019, indicated that the global prevalence of ASD ranges from 0.61% to 0.85%, surpassing the pooled prevalence of 0.35% observed in West Asian countries, including the Middle East (3, 4). Concerns have been expressed that underdiagnosis and underreporting of the condition in the region may be a contributing factor. In Iraq, no official estimate has been reported for the country as a whole. However, the northern region of Kurdistan has a reported prevalence of 0.89% (5).

Early detection of ASD is crucial, as early interventions have a positive impact on adaptive outcomes (6). While most children are diagnosed between the ages of 2 and 3, the variability in presentation has resulted in differences in the age at which most children receive a diagnosis (7). Therefore, it is essential for caregivers, usually parents, to detect the behaviours that might suggest the presence of ASD. Studies have been conducted to address awareness of autism’s core symptoms and attitudes toward children with ASD among parents in the general public. These studies aim to guide the implementation of educational campaigns that may lead to earlier recognition of ASD (8).

Following diagnosis, parents’ involvement plays an important role in treatment outcomes (9). Poor knowledge of available treatment options has been shown to delay treatment or lead to the endorsement of treatment options that have no proven efficacy (10). Caregivers are not simple bystanders; rather, they have an active role in decision-making, educational planning, and setting treatment goals. Additionally, most of the care tends to be provided at home, despite the presence of specialised healthcare centres (11, 12).

The Health Belief Model (HBM) provides a conceptual framework for how caregivers’ knowledge and attitudes might affect their behaviours regarding their child’s condition (13). According to this condition, health-related behaviour is triggered by a cue that might be either internal, such as the development of certain symptoms, or external, such as public health campaigns. The nature and extent of the cue needed to cause health-related behaviour will depend on their perceived susceptibility to the condition, their perceived severity, their perceived benefit of performing health-related behaviour, and the barriers against the behaviours.

Empirically, studies have shown that children whose parents have higher education levels and socioeconomic status have better treatment outcomes, while high parental stress is associated with worse outcomes (9). Educational programmes directed toward caregivers of children with ASD have shown a reduction in stress and increased confidence (14). Before such programmes can be developed and implemented, an evaluation of caregivers’ baseline knowledge and attitudes is necessary. This evaluation should also identify risk groups with lower knowledge to prioritise those groups during subsequent educational initiatives.

Caregivers’ attitudes can also greatly impact adaptive outcomes in children with ASD. Studies have shown that children from families with more negative attitudes tend to experience higher rates of behavioural and emotional issues, as well as reduced prosocial behaviour (15). Parenting styles that rely heavily on negative reinforcement and criticism have been associated with worse adaptive outcomes (16). Therefore, both caregivers’ knowledge and attitudes are often assessed simultaneously to address any negative attitudes during educational programmes.

In Iraq, literature surrounding ASD is scarce. According to a previous field report, specialised care centres are few and tend to be situated only around urban areas. In these low-resource settings, the role of caregivers becomes even more crucial. Few studies on caregivers’ knowledge and attitudes have been conducted in Iraq (17–19). These studies, however, often used small sample sizes, used questionnaires that did not cover aspects related to the aetiology and treatment of ASD, or in some cases reported only overall knowledge rather than individual item performance. Our study aims to assess caregivers’ knowledge of various aspects of ASD, including its aetiology, signs and symptoms, diagnosis, treatments, and outcomes in children. Additionally, we looked into negative attitudes towards mental health services, children with ASD themselves, and their educational needs.

## Methods

### Study design and setting

A multi-centre cross-sectional survey was conducted at Al-Sibtain Academy for Autism and Neurodevelopmental Disorders in Karbala and Baghdad’s National Centre for Autism and Child Psychiatry. These centres are responsible for providing specialised care for the majority of children with ASD in their respective provinces. They also provide care for children from surrounding provinces in the middle and southern regions of Iraq. Data was collected from caregivers attending with their children at the mentioned healthcare centres from April 1, 2024, to June 1, 2024. The entire study, including designing the study, collecting data, analysing it, and organising the final report, was conducted from February 17, 2024, to September 10, 2024.

This survey was part of a research collaboration between Al-Subtain University of Medical Sciences and the specified healthcare centres. The collaboration aimed to explore the epidemiological, clinical, and psychosocial aspects of children with ASD from Iraq through nine studies.

### Eligibility criteria

Only caregivers whose children received a confirmed diagnosis of ASD by a consultant in child and adolescent psychiatry based on the DSM-5 criteria were eligible to participate in our study. A caregiver was defined as an individual over the age of 18 who lives with the child and is responsible for meeting the child’s needs at home, including assistance with feeding, dressing, and using the toilet. Family members who accompanied the child but were not responsible for caregiving in the aforementioned areas were not included in our sample.

### Sample size requirement

A minimum sample size of 296 caregivers was required for this study, based on 26% as the percentage of caregivers with poor knowledge, as presented in an earlier study from Iraq (18), and 5% as the desired precision for the proportion estimate. The OpenEpi software ver. 3.01 was utilised to calculate the required sample size (20).

### Data collection tool

A structured questionnaire consisting of three sections (demographics, knowledge, and attitudes) was utilised in this study **(refer to research questionnaire)**. The first section enquired about both the caregiver’s and the child’s demographic characteristics. For the caregivers, eight items were included: age (in years), relation to the child, marital status, educational level, residence, caregiver-reported family income, the total number of children in the family, and the number of children with ASD. For the children, six items were included for age (years), gender, birth order, age at ASD diagnosis, and the presence and type of comorbid conditions.

To assess caregivers’ knowledge of ASD, a survey was developed based on the Autism Stigma and Knowledge Questionnaire (ASK-Q) and a review of the literature (17, 18, 21–25). The ASK-Q was originally created in 2017 to cover a wide range of content related to different aspects of ASD, including aetiology, symptoms/diagnosis, and treatment. Authors of the original validation study reported good construct validity and reliability, indicated by an intraclass correlation of 0.88 and Cronbach’s α of 0.86 (10). The ASK-Q has also shown cross-cultural applicability and has been translated and tested into different languages, including Mongolian (α = 0.72), Chinese (α = 0.89), and Arabic (α = 0.83) (26–29).

For each item in the survey, three answers were provided: “yes”, “no”, and “I don’t know”. The survey includes 48 items covering aetiology (13 items), signs and symptoms (23 items), diagnosis (2 items), treatment (6 items), and outcome (4 items). In accordance with the scoring system outlined for the ASK-Q items in the initial validation study, participants who answered correctly received a score of 1 on the item, whereas those who provided incorrect or neutral answers received a score of 0 (21). In total, knowledge scores were calculated by summing up the scores of all items, resulting in a total of forty-eight points.

Attitudes toward ASD were assessed across various domains using a survey developed, including items described in previous studies (21, 30–33). These domains included attitudes towards mental health services (2 items), attitudes towards children with ASD (6 items), attitudes towards the education of children with ASD (4 items), and attitudes towards parents’ responsibility (1 item). All items, except those related to attitudes towards mental health services, were presented on a 5-point Likert scale ranging from “strongly agree” to “strongly disagree”.

Two independent psychiatrists reviewed the questionnaire for content validity before data collection. In addition, 25 caregivers participated in a pilot study to test item clarity. Cronbach’s α showed that the knowledge scale is internally consistent at 0.73, higher than the recommended cut-off point of 0.70 (34). Due to a low Cronbach’s alpha, attitudes had no overall or domain-level scores, as items were heterogeneous and should be discussed individually.

### Data quality assurance

Multiple steps were taken to ensure data quality. Starting with the interview, all items were completed to reduce missing values. Second, to test intra-item validity, one item of the outcome knowledge domain was duplicated as a negative-form sentence to reduce random answer bias. Inconsistent respondents were excluded from the analysis. Data analysis began only after the completion of data collection. Finally, Equator Network’s STROBE cross-sectional studies guideline was used to mitigate reporting bias (35).

### Statistical analysis

Statistical analysis was conducted using the Statistical Package for Social Sciences (SPSS) program version 28, with a significance level set at <0.05.

Categorical variables were described with counts and proportions. To evaluate normality, both the D’Agostino-Pearson K2 and Shapiro-Wilk tests were used (**Table 1**), as the latter might be too sensitive to large sample sizes on its own. The mean and SD were used to describe normally distributed variables, while the median and IQR were employed for non-normal distributions. To aid comparison with recently published studies, caregivers were classified as having good knowledge if they answered >50% of questions correctly (score: ≥25) and poor knowledge otherwise (23, 36). The GraphPad Prism program version 10 was employed to visualise this data. The time since the child’s diagnosis was calculated by subtracting the child’s age at the time of diagnosis from their current age.

**Table 1:** Normality testing for the study variables.

Variables	Shapiro-wilk			D’Agostino-pearson K^2^	
	Statistic	df	P-value	Statistic	P-value
Age (Years)	0.957	302	**<0.001**	36.526	**<0.001**
No. of children	0.891	302	**<0.001**	54.451	**<0.001**
Child age (Years)	0.922	302	**<0.001**	48.168	**<0.001**
Birth order	0.826	302	**<0.001**	61.180	**<0.001**
Time since the diagnosis (Months)	0.840	302	**<0.001**	83.049	**<0.001**
Total knowledge score	0.992	302	0.085	2.652	0.266

Statistically significant association are highlighted with bold text.

The association between knowledge scores and demographic characteristics was assessed using various statistical tests, including the independent samples t-test, Mann-Whitney U-test, ANOVA, and Kruskal-Wallis test. The choice of test depended on the number of demographic subgroups and the normality of knowledge scores within each subgroup. The Benjamin-Hochberg procedure was used to account for multiple tests and maintain a 5% false discovery rate (37). *Post-hoc* pairwise analyses were conducted using Tukey’s HSD test following ANOVA, and Dunn-Bonferroni test following Kruskal-Wallis. Spearman’s rank correlation was used to study the correlations between continuous demographic variables and knowledge scores.

Results from *post-hoc* pairwise analyses showed that college-educated caregivers had higher knowledge than illiterate, primary school, and secondary school-educated caregivers. Similarly, caregivers from low-income backgrounds had less knowledge than those from average or high-income backgrounds. Hence, lack of tertiary education and low income were identified as risk factors for poor knowledge and used to compare the performance on individual knowledge items using Chi-square and Fisher exact tests. Multiple linear regression was also performed for further validation by including the dichotomous risk variables (lack of tertiary education and low income) as predictors and the knowledge score as an outcome variable. The squared zero-order correlation was reported to indicate the fraction of total variance in knowledge attributable to each predictor. Meanwhile, the squared partial correlation was utilised to signify the unique contribution of each predictor by presenting the proportion of residual variance attributed to a specific predictor after controlling for others.

To aid in interpreting attitude items, “agree” and “strongly agree” for items 3, 4, 6, 7, and 12 were reclassified as “negative” attitudes, while those for items 5, 8, 9, 10, 11, and 13 were reclassified as “positive” attitudes. The same reclassification was done for “disagree” and “strongly disagree”. Additionally, to explore the effect of the complexity of the child’s presentation, chi-square was utilised to test the association between the presence of comorbid conditions and items related to the attitudes of the parents.

## Results

### Data validation and inclusion

During the data collection period, 314 caregivers were interviewed. Of these, 12 (3.8%) were excluded due to inconsistent answers on the duplicated item used as a data validation procedure. In total, 302 (96.2%) caregivers were included in our sample: 180 (59.6%) from Al-Subtain Academy and 122 (40.4%) from Baghdad’s National Centre.

### Sample characteristics

Mothers comprised the highest percentage of caregivers (218, 72.2%), while fathers and other family members, such as the child’s sibling, grandparent, aunt, or uncle, accounted for 48 (15.9%) and 36 (11.9%), respectively. 12 (4.0%) of the caregivers were illiterate, and 60 (19.9%) came from a low-income background. 141 (46.7%) of the children in our study had other comorbid conditions, such as ADHD (80, 26.4%), intellectual issues (42, 13.9%), and cerebral palsy (9, 2.9%). Other demographic factors for both the caregivers and their children are summarised in **Table 2**.

**Table 2:** Sample characteristics (N = 302).

Characteristic	Distribution
Caregiver demographics	
Age (Median, IQR)	35 (30, 42)
Relation to the child (N, %)	
Mother	218 (72.2)
Father	48 (15.9)
Others	36 (11.9)
Marital status (N, %)	
Single	8 (2.6)
Married	273 (90.4)
Divorced/Widowed	21 (7.0)
Educational level (N, %)	
Illiterate	12 (4.0)
Primary school	56 (18.5)
Secondary school	90 (29.8)
Diploma	23 (7.6)
Bachelor or above	121 (40.1)
Caregiver-reported family income (N, %)	
Low	60 (19.9)
Average	196 (64.9)
High	46 (15.2)
Residence (N, %)	
Urban	281 (93.0)
Rural	21 (7.0)
Number of children (Median, IQR)	3 (2, 4)
Number of children with ASD (N, %)	
1	289 (95.7)
2	10 (3.3)
≥3	3 (10)
Child demographics	
Age (Years) (Median, IQR)	5 (3.6 – 7)
Time since the diagnosis (Years) (Median, IQR)	1.5 (0.5 – 3)
Birth order (Median, IQ)	2 (1 – 3)
Gender (N, %)	
Male	234 (77.5)
Female	68 (22.5)
Comorbidity (N, %)	
Yes	141 (46.7)
No	161 (53.3)

### Knowledge of ASD among caregivers

**Figure 1:**
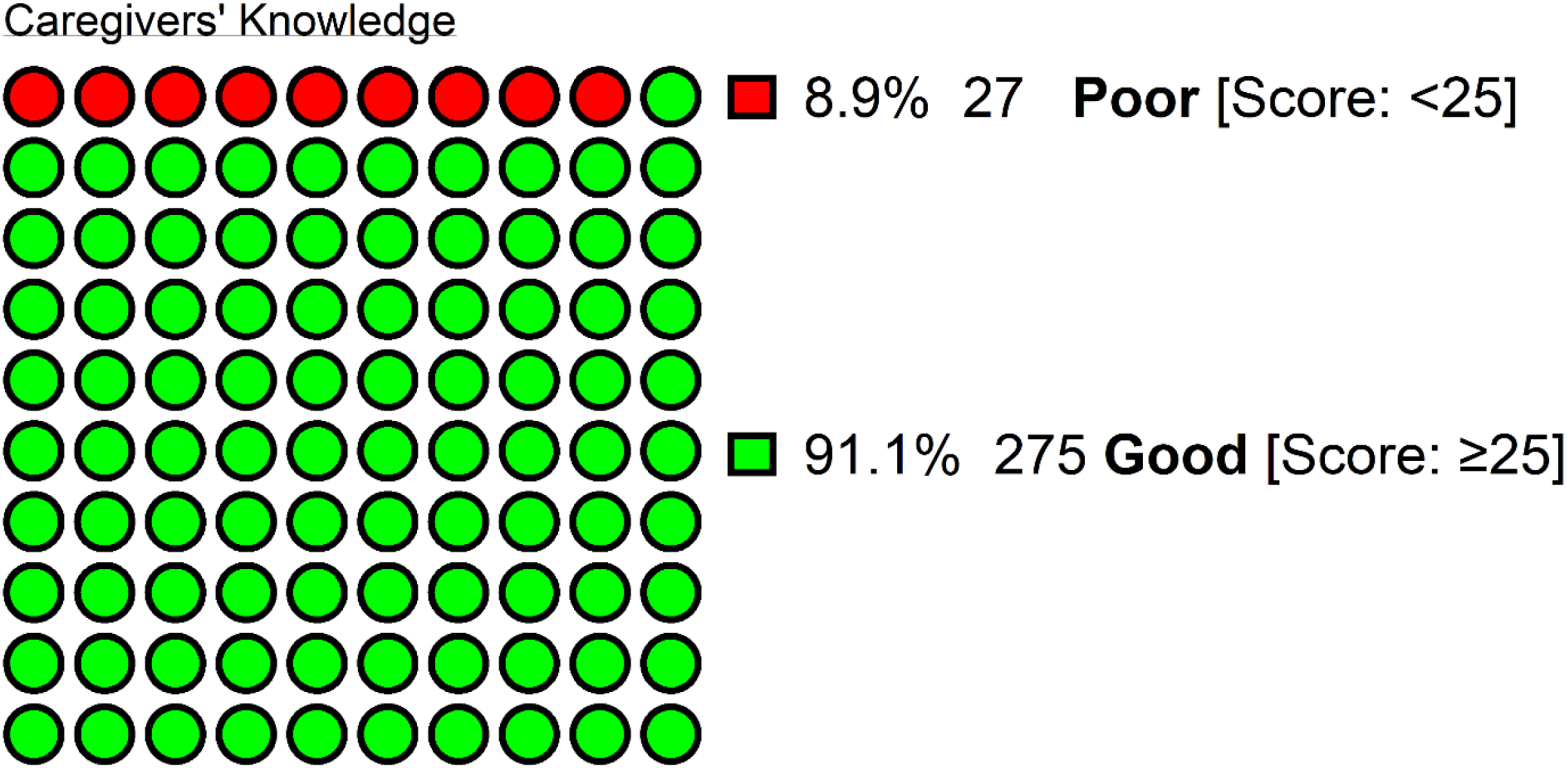
The knowledge of Iraqi caregivers toward autism spectrum disorder (N = 302), with good knowledge defined as having >50% of the total score on the 48-points knowledge scale.

In our sample, the average knowledge score was 31.7 (± 5.1) out of 48. Out of the caregivers surveyed, 27 (8.9%) had low knowledge and 275 (91.1%) had a good level of knowledge regarding ASD (**Figure 1**). The performance on individual knowledge items is summarised in **Table 3**.

**Table 3:** Knowledge among caregivers of children with autism spectrum disorder (N = 302).

Knowledge items	Correct responses N (%)	Incorrect responses N (%)
Aetiology		
Cold, rejecting parents can cause ASD	105 (34.8)	197 (65.2)
Excessively watching TV	45 (14.9)	257 (85.1)
Traumatic experiences in early life	104 (34.4)	198 (65.6)
ASD is a developmental condition	135 (44.7)	167 (55.3)
ASD is a communication condition	31 (10.3)	271 (89.7)
Unfavourable pregnancy can cause ASD	174 (57.6)	128 (42.4)
ASD occurs only in children	197 (65.2)	105 (34.8)
Genetic factors can cause ASD	186 (61.6)	116 (38.4)
Vaccination can cause ASD	186 (61.6)	116 (38.4)
ASD is more diagnosed in girls	185 (61.3)	117 (38.7)
ASD is more common among siblings	106 (35.1)	196 (64.9)
ASD has still unknown causes	228 (75.5)	74 (24.5)
ASD can be prevented	86 (28.5)	216 (71.5)
Signs and symptoms		
Strange reactions to sensory input	227 (75.2)	75 (24.8)
Trouble understanding facial expressions	208 (68.9)	94 (31.1)
Some children do not talk	277 (91.7)	25 (8.3)
Most children have the same symptoms	160 (53.0)	142 (47.0)
Trouble with loud noises	249 (82.5)	53 (17.5)
Need for routine and sameness	245 (81.1)	57 (18.9)
Intense interest in parts of objects	213 (70.5)	89 (29.5)
Problems with aggression or hyperactivity	225 (74.5)	77 (25.5)
Difficulty with everyday language	268 (88.7)	34 (11.3)
Repeating the words of the others	201 (66.6)	101 (33.4)
Delayed response to name	266 (88.1)	36 (11.9)
Excessively focused on one thing	257 (85.1)	45 (14.9)
Repeatedly spin objects or flap arms	261 (86.4)	41 (13.6)
Do not look at things when pointed at	211 (69.9)	91 (30.1)
No eye contact when spoken to	227 (75.2)	75 (24.8)
Do not enjoy the presence of others	198 (65.6)	104 (34.4)
May lose acquired speech	189 (62.6)	113 (37.4)
Failure to develop peer relationships	254 (84.1)	48 (15.9)
A social smile is absent	196 (64.9)	106 (35.1)
Loss of interest in their surrounding	192 (63.6)	110 (36.4)
Abnormal eating habits	236 (78.1)	66 (21.9)
Lack of will to share enjoyment	227 (75.2)	75 (24.8)
Lack of imaginative play	139 (46.0)	163 (54.0)
Diagnosis		
can be diagnosed as early as 18 months	169 (56.0)	133 (44.0)
Diagnosis is done by clinical observation	253 (83.8)	49 (16.2)
Modes of treatment		
Medications	213 (70.5)	89 (29.5)
Speech therapy	274 (90.7)	28 (9.3)
Occupational therapy	273 (90.4)	29 (9.6)
Nutritional therapy	217 (71.9)	85 (28.1)
Behavioural therapy is more effective	288 (95.4)	14 (4.6)
Medications can alleviate core symptoms	227 (75.2)	75 (24.8)
Outcome		
A complete cure is possible	197 (65.2)	105 (34.8)
Earlier treatment is more effective	284 (94.0)	18 (6.0)
Need extra help to learn	253 (83.8)	49 (16.2)
Most children outgrow ASD	47 (15.6)	255 (84.4)

A statistically significant association was demonstrated between caregivers’ knowledge of ASD and their educational level and family income (**Table 4**). Child comorbidity was significantly associated with higher knowledge but only before adjusting for multiple testing (adjusted p-value = 0.088). No statistically significant correlations were found with the parents’ age (adjusted p-value = 0.673) or the time since the diagnosis of ASD (adjusted p-value = 0.249) (**Table 5**).

**Table 4:** Association between overall knowledge score among parents of children with autism spectrum disorder and demographic factors (N = 302).

Demographic factors	Knowledge score	Test statistic	P-value	Adjusted P-value^e^
Relation to the child (Mean, SD)				
Mother	31.8 (26.6, 37.0)	1.518	0.221**^a^**	0.376
Father	32.6 (28.0, 37.2)			
Others	30.6 (26.0, 35.2)			
Marital status (Mean, SD)				
Single	31.5 (26.7, 36.3)	0.047	0.954**^a^**	0.960
Married	31.7 (26.6, 36.8)			
Divorced/Widowed	32.0 (27.2, 36.8)			
Educational level (Mean, SD)				
Illiterate	28.3 (25.0, 31.6)	6.222	8*10–^5 a^	**0.001**
Primary school	30.3 (25.8, 34.8)			
Secondary school	30.9 (25.8, 36.0)			
Diploma	32.5 (26.6, 38.4)			
Bachelor or above	33.2 (28.4, 38.0)			
Caregiver-reported family income (Median, IQR)				
Low	29.5 (27, 32)	13.635	**0.001^b^**	**0.006**
Average	33 (29, 36)			
High	33 (28, 38)			
Residence (Mean, SD)				
Urban	31.8 (26.7, 36.9)	1.151	0.251**^c^**	0.376
Rural	30.5 (25.6, 35.4)			
Child’s gender (Mean, SD)				
Male	32.0 (26.9, 37.1)	1.309	0.192**^c^**	0.376
Female	31.0 (26.1, 35.9)			
Child comorbidity (Median, IQR)				
Yes	33 (29, 36)	9616	**0.022^d^**	0.088
No	31 (28, 35)			

^a^One-way ANOVA test was used for analysis with 0.05 as the cut-off point for statistical significance.

^b^Kruskal-Wallis test was used for analysis with 0.05 as the cut-off point for statistical significance.

^c^Independent Samples T test was used for analysis with 0.05 as the cut-off point for statistical significance.

^d^Man-Whitney U test was used for analysis with 0.05 as the cut-off point for statistical significance.

^e^Benjamin-Hochberg procedure was used to adjust the P-value for multiple testing.

Statistically significant association are highlighted with bold text.

**Table 5:** Correlation between overall knowledge score among parents of children with autism spectrum disorder and demographic factors (N = 302).

Demographic factors	Knowledge score		
	Correlation coefficient^a^	P-value	Adjusted P-value^b^
Age (Years)	0.038	0.505	0.673
No. of children	-0.080	0.166	0.376
Child age (Years)	0.003	0.960	0.960
Time since the diagnosis (Years)	0.100	0.083	0.249
Birth order	0.010	0.866	0.960

^a^Spearman’s rank correlation was used for statistical analysis with 0.05 as a cut-off point for significance.

^b^Benjamin-Hochberg procedure was used to adjust the P-value for multiple testing.

Through *post-hoc* pairwise analysis, significant differences in knowledge scores were found between caregivers with a bachelor’s degree or higher and those who were illiterate (p-value = 0.009), had a primary school degree (p-value = 0.002), or had a secondary school degree (p-value = 0.007) (**Table 6**). Similarly, differences were identified between caregivers from low-income backgrounds and those from average (p-value = 0.002) or good backgrounds (p-value = 0.005), but not between individuals from average and good backgrounds (p-value = 1.000). Therefore, performance on individual knowledge items was compared between caregivers based on tertiary education attainment and low-income backgrounds (**Table 7**). When further investigated with multiple linear regression, lack of tertiary education had a significant positive relation, uniquely explaining 3.8% of the variance in knowledge scores (B = 2.177, p-value = 0.0006), while low family income was no longer significant after controlling for lack of tertiary education (B = 1.231, p-value = 0.119) (**Table 8**). These two predictors also showed a statistically significant association with one another, as pointed out by the fact that only 2 (3.3%) of those with low income had tertiary education compared to 142 (58.7%) of those with average or high family income (p-value <0.001) (**Table 9**).

**Table 6:** Pairwise analysis for the association between education levels and family income with knowledge score among parents of children with autism spectrum disorder (N = 302).

Variables	Pairwise grouping	P-value
Education level^a^	Illiterate – Primary school	0.714
	Illiterate – Secondary school	0.424
	Illiterate – Diploma	0.118
	Illiterate – Bachelor or above	**0.009**
	Primary school – Secondary school	0.947
	Primary school – Diploma	0.360
	Primary school – Bachelor or above	**0.002**
	Secondary school – Diploma	0.630
	Secondary school – Bachelor or above	**0.007**
	Diploma – Bachelor or above	0.969
Caregiver-reported family income^b^	Low – Average	**0.002**
	Low – High	**0.005**
	Average – High	1.000

^a^Tukey’s HSD test was used for pairwise analysis with 0.05 as a cut-off point for statistical significance.

^b^Dunn-Bonferroni test was used for pairwise comparison with 0.05 as a cut-off point for statistical significance.

Statistically significant association are highlighted with bold text.

**Table 7:** Knowledge item performance among risk groups in caregivers of autistic children (N = 302).

Items	Tertiary education				Low family income			
	No 158 (%)^a^	Yes 144 (%)^a^	P-value^b^	Adj. P-value^d^	Yes 60 (%)^a^	No 242 (%)^a^	P-value^b^	Adj. P-value^d^
Aetiology								
Cold, rejecting parents can cause autism	51 (32.3)	54 (37.5)	0.341	0.574	17 (28.3)	88 (36.4)	0.242	0.465
Excessively watching TV	19 (12.0)	26 (18.1)	0.443	0.595	11 (18.3)	34 (14.0)	0.420	0.650
Traumatic experiences in early life	50 (31.6)	54 (37.5)	0.285	0.537	23 (38.3)	81 (33.5)	0.478	0.695
Autism is a neurodevelopmental disorder	69 (43.7)	66 (45.8)	0.729	0.833	23 (38.3)	112 (46.3)	0.268	0.475
Autism is a communication disorder	19 (12.0)	12 (8.3)	0.291	0.537	10 (16.7)	21 (8.7)	0.068	0.272
Unfavourable pregnancy can cause autism	87 (55.1)	87 (60.4)	0.347	0.595	35 (58.3)	139 (57.4)	0.900	0.989
Autism occurs only in children	98 (62.0)	99 (68.8)	0.220	0.528	39 (65.0)	158 (65.3)	0.966	0.989
Genetic factors can cause autism	89 (56.3)	97 (67.4)	**0.049**	0.235	37 (61.7)	149 (61.6)	0.989	0.989
Vaccination can cause autism	99 (62.7)	87 (60.4)	0.689	0.807	37 (61.7)	149 (61.6)	0.989	0.989
Autism is more diagnosed in girls	91 (57.6)	94 (65.3)	0.171	0.483	31 (51.7)	154 (63.6)	0.088	0.305
Autism is more common among siblings	48 (30.4)	58 (40.3)	0.072	0.284	13 (21.7)	93 (38.4)	**0.015**	0.144
Autism has still unknown causes	116 (73.4)	112 (77.8)	0.379	0.595	45 (75.0)	183 (75.6)	0.920	0.989
Autism can be prevented	41 (25.9)	45 (31.3)	0.308	0.548	18 (30.0)	68 (28.1)	0.770	0.948
Signs and symptoms								
Strange reactions to sensory input	114 (72.2)	113 (78.5)	0.204	0.528	40 (66.7)	187 (77.3)	0.089	0.305
Trouble understanding facial expressions	106 (67.1)	102 (70.8)	0.534	0.675	42 (70.0)	166 (68.6)	0.833	0.989
Some children do not talk	143 (90.5)	134 (93.1)	0.422	0.596	51 (85.0)	226 (93.4)	**0.035**	0.210
Most children have the same symptoms	85 (53.8)	75 (52.1)	0.766	0.855	34 (56.7)	126 (52.1)	0.523	0.738
Trouble with loud noises	128 (81.0)	121 (84.0)	0.491	0.655	46 (76.7)	203 (83.9)	0.188	0.451
Need for routine and sameness	117 (74.1)	128 (88.9)	**0.0099**	0.095	45 (75.0)	200 (82.6)	0.176	0.451
Intense interest in parts of objects	104 (65.8)	109 (75.7)	0.060	0.262	38 (63.3)	175 (72.3)	0.172	0.451
Problems with aggression or hyperactivity	116 (73.4)	109 (75.7)	0.650	0.780	46 (76.7)	179 (74.0)	0.668	0.867
Difficulty with everyday language	134 (84.8)	134 (93.1)	**0.024**	0.165	52 (86.7)	216 (89.3)	0.570	0.782
Repeating the words of the others	94 (59.5)	107 (74.3)	**0.006**	0.095	33 (55.0)	168 (69.4)	0.062**^c^**	0.271
Delayed response to name	136 (86.1)	130 (90.3)	0.260	0.532	51 (85.0)	215 (88.8)	0.411	0.650
Excessively focused on one thing	129 (81.6)	128 (88.9)	0.077	0.284	49 (81.7)	208 (86.0)	0.404	0.650
Repeatedly spin objects or flap arms	136 (86.1)	125 (86.8)	0.853	0.910	48 (80.0)	213 (88.0)	0.105	0.330
Do not look at things when pointed at	111 (70.3)	100 (69.4)	0.878	0.915	47 (78.3)	164 (67.8)	0.110	0.330
No eye contact when spoken to	116 (73.4)	111 (77.1)	0.461	0.632	44 (73.3)	183 (75.6)	0.714	0.902
Do not enjoy the presence of others	99 (62.7)	99 (68.8)	0.266	0.532	37 (61.7)	161 (66.5)	0.478	0.695
May lose acquired speech	98 (62.0)	91 (63.2)	0.834	0.910	39 (65.0)	150 (62.0)	0.666	0.867
Failure to develop peer relationships	126 (79.7)	128 (88.9)	**0.030**	0.180	45 (75.0)	209 (86.4)	**0.031**	0.210
A social smile is absent	94 (59.5)	102 (70.8)	**0.039**	0.208	39 (65.0)	157 (64.9)	0.986	0.989
Loss of interest in their surrounding	97 (61.4)	95 (66.0)	0.409	0.595	34 (56.7)	158 (65.3)	0.214	0.452
Abnormal eating habits	123 (77.8)	113 (78.5)	0.896	0.915	50 (83.3)	186 (76.9)	0.277	0.475
Lack of will to share enjoyment	119 (75.3)	108 (75.0)	0.949	0.949	45 (75.0)	182 (75.2)	0.974	0.989
Lack of imaginative play	69 (43.7)	70 (48.6)	0.390	0.595	20 (33.3)	119 (49.2)	**0.028**	0.210
Diagnosis								
can be diagnosed as early as 18 months	77 (48.7)	92 (63.9)	**0.008**	0.095	29 (48.3)	140 (57.9)	0.184	0.451
Diagnosis is done by clinical observation	127 (80.4)	126 (87.5)	0.155**^c^**	0.471	44 (73.3)	209 (86.4)	**0.012**	0.144
Modes of treatment								
Medications	108 (68.4)	105 (72.9)	0.385	0.595	36 (60.0)	177 (73.1)	**0.046**	0.240
Speech therapy	145 (91.8)	129 (89.6)	0.512	0.664	52 (86.7)	222 (91.7)	0.226	0.452
Occupational therapy	144 (91.1)	129 (89.6)	0.647	0.780	51 (86.7)	221 (91.3)	0.273	0.475
Nutritional therapy	108 (68.4)	109 (75.7)	0.157	0.471	37 (61.7)	180 (74.4)	0.050	0.240
Behavioural therapy is more effective	145 (91.8)	143 (99.3)	**0.002**	**0.048**	53 (88.3)	235 (97.1)	**0.009^c^**	0.144
Medications can alleviate core symptoms	113 (71.5)	114 (79.2)	0.124	0.425	34 (56.7)	193 (79.8)	**0.0002**	**0.005**

Items	Tertiary education				Poor income			
	No 158 (%)^a^	Yes 144 (%)^a^	P-value^b^	Adj. P-value^d^	Yes 60 (%)^a^	No 242 (%)^a^	P-value^b^	Adj. P-value^d^
Outcome								
Complete cure is possible	90 (57.0)	107 (74.3)	**0.002**	**0.048**	27 (45.0)	170 (70.2)	**0.0002**	**0.005**
Earlier treatment is more effective	146 (92.4)	138 (95.8)	0.209	0.528	54 (90.0)	230 (95.0)	0.217**^c^**	0.452
Need extra help to learn	125 (79.1)	129 (88.9)	**0.021**	0.165	47 (78.3)	206 (85.1)	0.202	0.452
Most children outgrow autism	21 (13.3)	26 (18.1)	0.254	0.532	9 (15.0)	38 (15.7)	0.893	0.989

^a^Counts and column percent are described as individuals who have answered correctly on the indicated items.

^b^Chi-square test was used for statistical analysis with a cut-off point of 0.05 for statistical significance.

^c^Fisher exact test was used for statistical analysis with a cut-off point of 0.05 for statistical significance.

^d^Benjamin-Hochberg procedure was used to adjust the P-value for multiple testing.

Statistically significant association are highlighted with bold text.

**Table 8:** Multiple regression analysis model for caregivers’ knowledge, with lack of tertiary education and low family income as predictor variables.

Variables	Knowledge score					
	B	S.E	t	P-value	Co ^a^	UC ^b^
Constant	29.727	0.632	47.017	**<0.001**		
Tertiary education	2.177	0.629	3.463	**0.0006**	0.067	0.038
Low family income	1.231	0.787	1.564	0.119	0.037	0.008
**Adjusted R^2^ [P-value]**	0.068 (1*10^-5^)					

^a^Squared zero-order correlation was used to measure the contribution of individual predictors within the regression model.

^b^Squared partial correlation was used to measure the unique contribution of each individual predictor within the regression model.

Statistically significant association are highlighted with bold text.

**Table 9:** The association between lack of tertiary education and low family income among caregivers of children with autism spectrum disorder (N = 302).

Tertiary education	Low family income		
	No 242 (%)	Yes 60 (%)	P-value^a^
No	100 (41.3)	58 (96.7)	1.5*10^-14^
Yes	142 (58.7)	2 (3.3)	

^a^Chi-square test was used for statistical analysis with a cut-off point of 0.05 for statistical significance.

Statistically significant association are highlighted with bold text.

### Attitudes toward ASD among caregivers

Of the caregivers surveyed, 277 (91.7%) stated that they would visit the closest health organisation if their child could not speak by 2 years of age, while only 8 (2.6%) answered that there is no need to worry. The most consulted specialists were psychiatrists (139, 46.0%), followed by children’s primary healthcare physicians (42, 13.9%) and neurologists (41, 13.6%). Caregivers’ attitudes on various aspects of ASD are shown in **Table 10**. The item related to the responsibility of parents towards their children with special needs received the highest number of positive responses, with 285 (94.4%). Conversely, the lowest proportion of positive attitudes was towards the notion that children with ASD are deliberately negativistic and non-compliant, with only 105 (34.8%) responses. Parents of children with comorbid conditions were more likely to feel ashamed of the child’s diagnosis. However, this difference was only significant before adjusting for multiple testing (adjusted p-value = 0.286).

**Table 10:** The association between the presence of comorbid conditions and the parents’ attitudes toward children with autism spectrum disorder (N = 302).

Attitudes toward children with ASD	The presence of comorbid conditions					
	No 161 (%)^a^	Yes 141 (%)^a^	Total 302 (%)^a^	Test statistic	p-value^b^	Adjusted p-value^c^
Feeling ashamed of my child’s diagnosis						
Positive attitude	108 (67.1)	74 (52.5)	182 (60.3)	7.302	**0.026**	0.286
Neutral	9 (5.6)	8 (5.7)	17 (5.6)			
Negative attitude	44 (27.3)	59 (41.8)	103 (34.1)			
All children with ASD have problems with aggression						
Positive attitude	111 (68.9)	89 (63.1)	200 (66.2)	1.469	0.480	0.662
Neutral	27 (16.8)	31 (22.0)	58 (19.2)			
Negative attitude	23 (14.3)	21 (14.9)	44 (14.6)			
Children with ASD can live independently						
Positive attitude	70 (51.1)	72 (51.1)	142 (47.0)	1.739	0.419	0.662
Neutral	59 (36.6)	45 (31.9)	104 (34.4)			
Negative attitude	32 (19.9)	24 (17.0)	56 (18.5)			
Children with ASD always have a severe disability						
Positive attitude	77 (47.8)	59 (41.8)	136 (45.0)	1.230	0.541	0.662
Neutral	54 (33.5)	55 (39.0)	109 (36.1)			
Negative attitude	30 (18.6)	27 (19.1)	57 (18.9)			
Children with ASD are deliberately negativistic and non-compliant						
Positive attitude	62 (38.5)	43 (30.5)	105 (34.8)	5.437	0.066	0.363
Neutral	48 (29.8)	35 (24.8)	83 (27.5)			
Negative attitude	51 (31.7)	63 (44.7)	114 (37.7)			
Most children with ASD have special talents						
Positive attitude	109 (67.7)	90 (63.8)	199 (65.9)	0.520	0.771	0.771
Neutral	18 (11.2)	17 (12.1)	35 (11.6)			
Negative attitude	34 (21.1)	34 (24.1)	68 (22.5)			
Children with ASD should receive special education						
Positive attitude	131 (81.4)	110 (78.0)	241 (79.8)	2.806	0.246	0.662
Neutral	11 (6.8)	6 (4.3)	17 (5.6)			
Negative attitude	19 (11.8)	25 (17.7)	44 (14.6)			
Children with ASD should be enrolled in normal classes						
Positive attitude	118 (73.3)	109 (77.3)	227 (75.2)	1.226	0.542	0.662
Neutral	19 (11.8)	17 (12.1)	36 (11.9)			
Negative attitude	24 (14.9)	15 (10.6)	39 (12.9)			
Children with ASD are more intelligent than indicated by appropriate tests						
Positive attitude	115 (71.4)	103 (73.0)	218 (72.2)	0.685	0.710	0.771
Neutral	24 (14.9)	23 (16.3)	47 (15.6)			
Negative attitude	22 (13.7)	15 (10.6)	37 (12.3)			
Children with ASD are unable to pursue a university education						
Positive attitude	73 (45.3)	54 (38.3)	127 (42.1)	1.671	0.434	0.662
Neutral	60 (37.3)	57 (40.4)	117 (38.7)			
Negative attitude	28 (17.4)	30 (21.3)	58 (19.2)			
Parents are responsible for obtaining services for their children with special needs						
Positive attitude	152 (94.4)	133 (94.3)	285 (94.4)	1.675	0.533**^d^**	0.662
Neutral	7 (4.3)	8 (5.7)	15 (5.0)			
Negative attitude	2 (1.2)	0 (0.0)	2 (0.7)			

^a^Counts and column percentages were used to describe the data distribution.

^b^Chi-square test was used for statistical analysis with a cut-off point of 0.05 for statistical significance.

^c^Benjamin-Hochberg procedure was used to adjust the P-value for multiple testing.

^d^Fisher exact test was used for statistical analysis with a cut-off point of 0.05 for statistical significance.

## Discussion

Less than one-tenth (8.9%) of the caregivers demonstrated poor knowledge. This percentage is lower than the 26% reported in a previous study from Hilla province in Iraq. However, details regarding the specific cut-off point used were not mentioned (18). Recently published studies from Egypt and Nigeria also found that 68.3% and 98.7% of caregivers, respectively, had poor/unsatisfactory knowledge using the same cut-off point (<50% of the total score) (23, 36). These results are encouraging, as previous studies have shown that increasing caregivers’ knowledge can reduce stress, improve parenting competency, and lead to better adaptive outcomes in children (9, 14, 23, 38). The importance of conducting such programmes is also highlighted by the fact that, in our study, the time since the diagnosis of the child with ASD did not result in a significant increase in knowledge. Therefore, in our local setting, parents may not naturally gain more knowledge without support from a formal educational program.

Caregivers’ knowledge was found to be linked to their level of education and self-reported family income. A pairwise analysis of this connection identified caregivers without tertiary education and those from low-income backgrounds as at-risk groups for lower knowledge. This is consistent with previous results from Basra province in Iraq and Egypt, where the largest difference in knowledge was observed between caregivers with a college education and those without (17, 23). A study from China also identified the absence of tertiary education and a low family income as risk factors for lower knowledge (25). The relation between caregivers’ education and family income is well documented in the literature (39). This is also evident in our sample, where the relationship between family income and knowledge was no longer significant after controlling for tertiary education in multiple regression. This suggests that family income may not be independently connected to knowledge of ASD; rather, its significance stems from its relationship with the caregivers’ level of education.

Contrary to previous findings from Iraq, Egypt, and China, the association between parents’ age and knowledge in our study was not significant (19, 23, 25). Another study from Malaysia also found that mothers had more knowledge than fathers (22), while we found that both parents had comparable knowledge scores. This might be a result of the higher involvement of Arabic fathers in childcare or due to the fact that data collection involved only parents who attended with their children to the healthcare centres. These parents may have similar levels of involvement in their children’s care, leading to equal knowledge regardless of their biological roles. This idea could also account for the demonstrated similarity in knowledge regardless of marital status. Future studies should include both parents for each child to further explore these possibilities.

Caregivers of children with comorbid conditions, such as ADHD and intellectual issues, had higher knowledge scores. This could be attributed to more frequent contact with healthcare providers. However, this association was no longer significant after adjusting for p-values, so it cannot be definitively established (40).

The majority of caregivers (85.1%) believed that ASD can be caused by excessive TV watching. Additionally, 65.2% believed that cold-rejecting parents can cause ASD, and 38.4% thought that vaccines could lead to ASD. These misconceptions were also present in previous studies conducted in Iraq. For example, a study from 2020 reported prevalence rates of 64%, 38%, and 34.8% for these misconceptions, respectively. Another study from 2022 found that 67.3% of caregivers believed that emotional deprivation could cause the condition (18, 19). These misconceptions relate to the perceived susceptibility construct of the Health Belief Model and have been shown to affect caregivers’ health-related behaviour regarding their children by increasing vaccine refusal, stigmatisation, and self-blame among caregivers (41, 42). As such, the persistence of these misconceptions, despite previously published results indicating their prevalence, highlights the importance of conducting educational interventions to address them.

A third of the caregivers in our sample expressed feeling shame about their child’s diagnosis. Parents’ shame may also arise when children with ASD exhibit behaviours that are considered socially unacceptable in public (43). Hence, the complexity of the child’s presentation, including the presence of comorbid conditions, could worsen parents’ shame. In our study, the statistical link between parental shame and comorbid conditions was only evident before adjusting for multiple testing. Therefore, additional research is needed before drawing any definitive conclusion.

Caregivers, in our study, had a better perceived severity of ASD compared to perceived susceptibility. This can be shown through better performance on items related to ASD symptoms than aetiology, presumably due to exposure to the symptoms through their children or by talking to healthcare professionals (44). Specifically, 88.1% of caregivers identified delayed response to name as a potential symptom, 75.2% recognised poor eye contact, and 86.4% recognised repetitive and restrictive behaviours. These results align with a study from Hilla but are higher than those described in a study from Egypt, where only 28.3% recognised poor eye contact and 20% recognised repetitive and restrictive behaviours (18, 23).

Most caregivers perceived the benefits of behavioural therapy and early treatment for ASD. However, around a third (34.8%) still believed that a complete cure is possible, especially those without tertiary education (43.0%) or from low-income backgrounds (55.0%). Additionally, 84.4% thought that most children will outgrow ASD. These misconceptions need to be addressed, particularly among the subgroups mentioned earlier, as they may affect health-related behaviours in terms of treatment expectations and cooperation with healthcare professionals. Belief in a complete cure might lead parents to go through dangerous, unproven treatments, diverting attention from useful evidence-based supportive therapy. In 2019, the USA Food and Drug Administration published an article advising parents against falling prey to deceptive cure claims and highlighting how to identify such claims (45).

Most caregivers also agreed that caring for children with disabilities is the parents’ responsibility. However, many harboured negative attitudes, including the belief that all children with ASD are aggressive, severely disabled, intentionally negative, and non-compliant. These negative attitudes were present in similar proportions regardless of the child’s presentation complexity, defined by the presence of comorbid conditions. These stigmatising attitudes should be addressed through comprehensive educational campaigns, as they can result in caregivers perceiving their child’s behaviour or a disability as a barrier to accessing important health services (46). This occurs when caregivers do not pursue interventions and rather hide their child away to avoid social stigma.

Three-fourths of caregivers supported the inclusion of children with ASD in regular classes, consistent with previous studies from Iraq and Spain (18, 44). In the latter study, parents of typically developing children also had positive attitudes, albeit less so. Mainstream schools could provide an inclusive environment for children with ASD to interact with their peers, ultimately reducing social segregation and improving employment prospects (44). However, efforts to integrate these children into mainstream schools should be approached cautiously, taking into consideration the attitudes of typically developing peers, their families, and the broader cultural context. Previous studies have highlighted bullying as a common reason for families of children with ASD to change schools, underscoring the importance of addressing these concerns (47).

### Strengths and limitations

In this study, we explored the knowledge and attitudes among caregivers of autistic children, utilising a pairwise analytical approach to identify the group of caregivers who had low knowledge and negative attitudes toward children with ASD.

Certain limitations, however, can be demonstrated. Firstly, knowledge and attitudes were only assessed among caregivers who attended the healthcare centres. This sampling bias might result in an underestimation of the frequency of poor knowledge and negative attitudes, as these caregivers may possess more awareness due to their higher involvement in their children’s care. Additionally, lower generalisability might occur due to the exclusion of fathers and other family members who are less likely to attend healthcare centres with the children despite being responsible for a crucial portion of the childcare. To provide a more comprehensive depiction of caregivers’ knowledge and attitudes, this sampling bias should be addressed in future studies by involving other family members who may play a more minor, although crucial, part of providing care for the child. Secondly, data was collected from the middle and southern regions of Iraq and, as such, might not be representative of caregivers on a national level. Thirdly, the data collection tool utilised in this study might be subject to self-desirability bias, similar to other self-reported measures. Finally, an individual-item approach was adopted to discuss attitudes toward ASD. This was necessary based on the unfavourable internal consistency of attitude items. However, as a limitation, this causes an inability to explore overall attitudinal trends.

## Conclusion

In this study, we have shown that, despite the majority of caregivers having good overall knowledge of ASD, some misconceptions have persisted since earlier studies. Many of these misconceptions were related to the aetiology of ASD, namely the beliefs that ASD could be caused by vaccines, television, and negligent parenting. Given their persistence, more extensive educational interventions may be necessary. These interventions should also address treatment expectations and emphasise the lifelong nature of ASD, especially among caregivers without tertiary education and those from low-income backgrounds. Efforts should be made to reduce negative attitudes towards these children, such as the belief that they are always aggressive, intentionally negative, and non-compliant. Positive attitudes toward including children with ASD in mainstream schooling were also recognised; however, further studies are needed to evaluate other factors that may affect the children’s experience and adaptive outcomes.

## Data Availability

The datasets presented in this study can be found in online repositories. The names of the repository/repositories and accession number(s) can be found below: https://doi.org/10.17632/b7dc3ww9b7.1.
