# Supplementary material for: Risk factors for low knowledge and negative attitudes among caregivers of children with autism spectrum disorder in Iraq: a multi-centre cross-sectional study
Source: Front Psychiatry. 2025 Aug 4;16:1568467. doi: 10.3389/fpsyt.2025.1568467 (PMC12360076; doi:10.3389/fpsyt.2025.1568467)
Supplement: Supplementary file 1 [file Table1.docx]

**Research questionnaire (English version)**

**First:** Caregiver demographics

| **Age (years)** |  |
| --- | --- |
| **Relationship to the child** | 🞎 Father 🞎 Mother 🞎 other (mention) |
| **Marital status** | 🞎 Married 🞎 Divorced/widowed 🞎Single |
| **Educational level** | 🞎 Illiterate  🞎 Elementary school  🞎 Middle school  🞎 High school  🞎 Diploma  🞎 Bachelor  🞎 Masters  🞎 Ph.D. |
| **Residency** | 🞎 Urban 🞎 Rural |
| **Family income** | 🞎 Low 🞎 Average 🞎 High |
| **No. of children in the family** |  |
| **No. of auttistic children in the family** |  |

**Second:** Child demographics

| **Age (years)** |  |
| --- | --- |
| **Gender** | 🞎 Male 🞎 Female |
| **Birth order** |  |
| **Age at the time of diagnosis with autism (years)** |  |
| **Presence of comorbidity** | 🞎 No 🞎 Yes  **If yes, mention it:** |

**Knowledge**

1. Regarding the etiology of autism, please select the correct answer

|  | **Yes** | **I don’t know** | **No** |
| --- | --- | --- | --- |
| Autism is caused by **cold-rejecting parents** | 🞎 | 🞎 | 🞎 |
| Autism is caused by **excessively watching TV** | 🞎 | 🞎 | 🞎 |
| **Traumatic experiences** very early in life can cause autism | 🞎 | 🞎 | 🞎 |
| Autism is a **neurodevelopmental disorder** | 🞎 | 🞎 | 🞎 |
| Autism is a **communication disorder** | 🞎 | 🞎 | 🞎 |
| **Unfavorable circumstances** during the first six months of pregnancy | 🞎 | 🞎 | 🞎 |
| Autism is **present only in children** | 🞎 | 🞎 | 🞎 |
| **Genetic factors** play an important role in the development of autism | 🞎 | 🞎 | 🞎 |
| **Vaccines** can cause autism | 🞎 | 🞎 | 🞎 |
| Autism is more commonly diagnosed in **girls** | 🞎 | 🞎 | 🞎 |
| Children who have **autistic siblings** are more likely to develop autism | 🞎 | 🞎 | 🞎 |
| The cause of autism is **not yet known** for sure. | 🞎 | 🞎 | 🞎 |
| Autism is **preventable** | 🞎 | 🞎 | 🞎 |

1. Regarding symptoms of autism, please select the correct answer**:**

|  | **Yes** | **I don’t know** | **No** |
| --- | --- | --- | --- |
| Autistic children may have **strange reactions** to the way things smell, taste, look, feel, or sound. | 🞎 | 🞎 | 🞎 |
| Many Autistic children have **trouble understanding facial expressions** | 🞎 | 🞎 | 🞎 |
| Some Autistic children **do not talk** | 🞎 | 🞎 | 🞎 |
| Most Autistic children have the **same symptoms** | 🞎 | 🞎 | 🞎 |
| Many Autistic children have **trouble tolerating loud noises or certain types of touch** | 🞎 | 🞎 | 🞎 |
| Many Autistic children show **the need for routines and sameness.** (get **upset if their routine is changed).** | 🞎 | 🞎 | 🞎 |
| Some Autistic children **show intense interest in parts of objects.** | 🞎 | 🞎 | 🞎 |
| A lot of Autistic children have **problems with**  **being aggressive or hyperactive** | 🞎 | 🞎 | 🞎 |
| Many Autistic children have **difficulty using everyday language** to communicate their needs | 🞎 | 🞎 | 🞎 |
| **Repeating the word** of the others | 🞎 | 🞎 | 🞎 |
| **Delayed response to name** | 🞎 | 🞎 | 🞎 |
| Many times, Autistic children get **excessively focused on one thing** | 🞎 | 🞎 | 🞎 |
| Many Autistic children **repeatedly spin objects or flap their arms.** (having repetitive movements) | 🞎 | 🞎 | 🞎 |
| Most Autistic children may **not look at things when you point at them.** | 🞎 | 🞎 | 🞎 |
| Not being able to look into **the eyes of the person speaking to him** | 🞎 | 🞎 | 🞎 |
| Autistic children **do not enjoy the presence of others** | 🞎 | 🞎 | 🞎 |
| Some Autistic children **may lose acquired speech** | 🞎 | 🞎 | 🞎 |
| **Failure to develop peer relationships** appropriate for developmental age | 🞎 | 🞎 | 🞎 |
| **Social smile is usually absent** | 🞎 | 🞎 | 🞎 |
| **Loss of interest** in their surroundings | 🞎 | 🞎 | 🞎 |
| **Abnormal eating habits** | 🞎 | 🞎 | 🞎 |
| **Lack of spontaneous will to share enjoyment, interest, or activity with others** | 🞎 | 🞎 | 🞎 |
| **Lack of** imaginative play | 🞎 | 🞎 | 🞎 |
| Autism can be **diagnosed as early as 18 months** | 🞎 | 🞎 | 🞎 |

1. The diagnosis of autism is achieved through (select one)**:**

| **Blood tests** | **Genetic tests** | **Clinical observation** | **Radiological tests** | **I don’t know** |
| --- | --- | --- | --- | --- |
| 🞎 | 🞎 | 🞎 | 🞎 | 🞎 |

1. Treatment of autism includes the following (you can select more than one)**:**

| **Medications** | **Speech therapy** | **Occupational therapy** | **Nutritional therapy** |
| --- | --- | --- | --- |
| 🞎 | 🞎 | 🞎 | 🞎 |

1. Regarding the treatment of autism, select the correct answer**:**

|  | **Yes** | **I don’t know** | **No** |
| --- | --- | --- | --- |
| We now have treatments that can **cure autism** | 🞎 | 🞎 | 🞎 |
| The earlier treatment of autism starts, the **more effective** it tends to be* | 🞎 | 🞎 | 🞎 |
| **Behaviour therapy** is an intervention most likely to be effective for Autistic children | 🞎 | 🞎 | 🞎 |
| Autistic children **need extra help to learn** | 🞎 | 🞎 | 🞎 |
| With the proper treatment, most autistic children can eventually **outgrow** autism | 🞎 | 🞎 | 🞎 |
| Earlier treatment demonstrates **no additional benefit to Autistic children*** | 🞎 | 🞎 | 🞎 |
| **Medications** can alleviate core symptoms of autism | 🞎 | 🞎 | 🞎 |

*These items are used to check the validity of responses, one item should be retained during scoring

**Attitudes**

| 1) What will you do if you find out your **kids can not speak and make eye contact** with you by 2 years old?  A) Visit the closest healthcare organization  B) Wait and see  C) No need to worry  D) Don’t know | | | | | |
| --- | --- | --- | --- | --- | --- |
| 2) If you select to go to consult, which specialist would you like to consult for diagnostic evaluation?  A) Psychiatrist  B) Neurologist  C) Pediatrician  D) Primary health childcare physician  E) Psychotherapist  F) Physician from another specialty  G) Don’t know | | | | | |
|  | **Strongly**  **Agree** | **Agree** | **Neutral** | **Disagree** | **Strongly**  **disagree** |
| 3) I feel **ashamed** of my child's diagnosis | 🞎 | 🞎 | 🞎 | 🞎 | 🞎 |
| 4) All Autistic children usually have **problems with aggression** | 🞎 | 🞎 | 🞎 | 🞎 | 🞎 |
| 5) Autistic children can grow up to **live independently** | 🞎 | 🞎 | 🞎 | 🞎 | 🞎 |
| 6) Autism always causes **severe disability** | 🞎 | 🞎 | 🞎 | 🞎 | 🞎 |
| 7) Autistic children are deliberately **negativistic and non-compliant** | 🞎 | 🞎 | 🞎 | 🞎 | 🞎 |
| 8) Most Autistic children have **special talents** | 🞎 | 🞎 | 🞎 | 🞎 | 🞎 |
| 9) It is important that autistic children receive **special education** services at school | 🞎 | 🞎 | 🞎 | 🞎 | 🞎 |
| 10) Children with special needs **should be integrated** in to mainstream school | 🞎 | 🞎 | 🞎 | 🞎 | 🞎 |
| 11) Autistic children are **more intelligent** than scores from appropriate tests indicate | 🞎 | 🞎 | 🞎 | 🞎 | 🞎 |
| 12) Autistic children will be **unable to pursue** education at the university level | 🞎 | 🞎 | 🞎 | 🞎 | 🞎 |
| **13) Parents are responsible** for obtaining services for their own children with special need | 🞎 | 🞎 | 🞎 | 🞎 | 🞎 |

**Research questionnaire (Arabic version)**

**معلومات تخص الذي يقوم بملىء الاستمارة**

|  | **العمر (بالسنوات)** |
| --- | --- |
| اخرى (تذكر)🞎 الام 🞎 الأب 🞎 | **العلاقة بالطفل** |
| مطلق/ارمل 🞎 متزوج 🞎اعزب🞎 | **الحالة الزوجية** |
| 🞎 لا يعرف القراءة و الكتابه  🞎أبتدائى 🞎 متوسطة  🞎 اعدادي 🞎 دبلوم  🞎 بكلوريوس 🞎 ماجستير  🞎 دكتوراه | **المستوى التعليمي** |
| الريف 🞎 المدينة 🞎 | **محل الاقامة** |
| جيد🞎 متوسط 🞎 قليل 🞎 | **دخل الاسرة** |
|  | **عدد الأطفال في العائلة** |
|  | **عدد الأطفال المصابين باضطراب طيف التوحد في العائلة** |

**معلومات تخص الطفل**

|  | **العمر (بالسنوات)** |
| --- | --- |
| انثى 🞎 ذكر 🞎 | **الجنس** |
|  | **الترتيب في الأسرة** |
|  | **العمر عند التشخيص باضطراب طيف التوحد (بالسنوات)** |
| نعم 🞎 كلا 🞎  *اذا كان الجواب نعم **ما نوعه:** | **هل يعاني الطفل من أي مرض مصاحب؟** |

**فيما يتعلق بمسببات التوحد .، يرجى تحديد الإجابة الصحيحة:**

|  | **نعم** | **لا أعلم** | **لا** |
| --- | --- | --- | --- |
| التوحد ناتج عن **البرود ورفض الوالدين** |  |  |  |
| **تزيد مشاهدة التلفزيون** بشكل مفرط من فرص إصابة الأطفال بالتوحد |  |  |  |
| **التجارب المؤلمة** في وقت مبكر جدا من الحياة يمكن أن تسبب التوحد |  |  |  |
| التوحد هو **اضطراب في تطور العصبي الطفل** |  |  |  |
| التوحد هو**اضطراب في التواصل** |  |  |  |
| الظروف غير الملائمة **خلال الأشهر الست الأولى** من الحمل |  |  |  |
| التوحد **موجود فقط عند الأطفال** |  |  |  |
| تلعب **العوامل الوراثية** دورا مهما في حدوث مرض التوحد |  |  |  |
| **اللقاحات** يمكن أن تسبب التوحد |  |  |  |
| يتم تشخيص التوحد بشكل أكثر شيوعا عند **الفتيات** |  |  |  |
| الأطفال الذين لديهم **أشقاء مصابون بالتوحد** هم أكثر عرضة للإصابة بالتوحد |  |  |  |
| سبب التوحد **غير معروف بعد** على وجه اليقين. |  |  |  |
| يمكن **الوقاية**  من التوحد |  |  |  |

**فيما يتعلق بأعراض التوحد ، يرجى تحديد الإجابة الصحيحة:**

|  | **نعم** | **لا أعلم** | **لا** |
| --- | --- | --- | --- |
| قد يكون لدى الأطفال المصابين بالتوحد **ردود فعل غريبة** لرائحة لأشياء أو طعمها أو شكلها أو ملمسها أو صوتها. |  |  |  |
| يعاني العديد من الأطفال المصابين بالتوحد **من صعوبة في فهم تعابير الوجه** |  |  |  |
| بعض الأطفال المصابين بالتوحد **غير قادرين على الكلام** |  |  |  |
| معظم الأطفال المصابين بالتوحد لديهم **نفس الأعراض** |  |  |  |
| يعاني العديد من الأطفال المصابين بالتوحد من **صعوبة في تحمل الضوضاء العالية أو أنواع معينة من اللمس** |  |  |  |
| يظهر العديد من الأطفال المصابين **بالتوحد الحاجة إلى البقاء على نفس الروتين.** (ينزعجون إذا تم تغيير روتينهم) |  |  |  |
| يظهر بعض الأطفال المصابين **بالتوحد اهتماما شديدا بأجزاء من الأشياء** (مثل عجلات السيارات) |  |  |  |
| يعاني الكثير من الأطفال المصابين بالتوحد **من العدوانية (أن يكون الطفل عدواني) أو مفرط النشاط** |  |  |  |
| يواجه العديد من الأطفال المصابين بالتوحد **صعوبة في استخدام اللغة اليومية** للتعبير عن احتياجاتهم |  |  |  |
| **تكرار كلام الآخرين** |  |  |  |
| **تأخرالردعلى الاسم (عند مناداة الطفل باسمه)** |  |  |  |
| في كثير من الأحيان ، يركز الأطفال المصابون **بالتوحد بشكل مفرط على شيء واحد** |  |  |  |
| يقوم العديد من الأطفال المصابين **بالتوحد بتدوير الأشياء بشكل متكرر أو رفرفة أذرعهم.** ( **لديهم حركات تكراريه)** |  |  |  |
| قد لا ينظر معظم الأطفال المصابين **بالتوحد إلى الأشياء عندما تشير إليها.** |  |  |  |
| عدم القدرة على النظرفي**عيون الشخص** الذي يتحدث إليه |  |  |  |
| الأطفال المصابون بالتوحد **لا يستمتعون بوجود الآخرين** |  |  |  |
| قد يفقد بعض الأطفال المصابين **بالتوحد الكلام المكتسب** |  |  |  |
| **الفشل في تطوير علاقات الأقران** (الأصدقاء)المناسبة لعمر النمو |  |  |  |
| عادة ما تكون **الابتسامة الاجتماعية غائبة** |  |  |  |
| **فقدان الاهتمام بمحيطهم** |  |  |  |
| توجد لديهم **عادات الأكل غير الطبيعية** |  |  |  |
| **عدم وجود إرادة ذاتيه لمشاركة المتعة أو الاهتمام أو النشاط مع الآخرين** |  |  |  |
| **قلة اللعب التخيلي** |  |  |  |
| يمكن تشخيص التوحد **في وقت مبكر من 18 شهرا** (اقل من عمر السنتين) |  |  |  |

**يتم تشخيص التوحد من خلال (اختر واحدا):**

| **اختبارات الدم** | **الاختبارات الجينية** | **الملاحظة السريرية**  **للطفل** | **الاختبارات الإشعاعية** | **لا أعلم** |
| --- | --- | --- | --- | --- |
|  |  |  |  |  |

**يشمل علاج التوحد ما يلي (يمكنك اختيار أكثر من واحد):**

| **الادويه** | **علاج النطق** | **العلاج الوظيفي** | **العلاج الغذائي** |
| --- | --- | --- | --- |
|  |  |  |  |

**فيما يتعلق بعلاج التوحد ، حدد الإجابة الصحيحة:**

|  | **نعم** | **لا أعلم** | **لا** |
| --- | --- | --- | --- |
| لدينا الآن علاجات يمكن أن **تعالج التوحد بشكل كامل** |  |  |  |
| كلما بدا علاج التوحد مبكرا , كلما **زادت فعاليته*** |  |  |  |
| العلاج السلوكي هو تدخل من **المرجح أن يكون فعالا** للأطفال المصابين بالتوحد |  |  |  |
| يحتاج الأطفال المصابون بالتوحد إلى **مساعدة إضافية للتعلم** |  |  |  |
| مع العلاج المناسب ، يمكن لمعظم الأطفال المصابين بالتوحد في نهاية المطاف **تجاوز مرض التوحد** |  |  |  |
| العلاج المبكر **لا يظهر أي فائدة إضافية للأطفال المصابين بالتوحد*** |  |  |  |
| **الأدوية** يمكن أن تخفف من الأعراض الأساسية للتوحد |  |  |  |

**المواقف**

| ماذا ستفعل إذا اكتشفت أن طفلك **لايستطيع التحدث والتواصل بالعين معك** بعمر 2 سنة؟  1) زيارة اقرب مؤسسه صحية  2) انتظروانظر 3) لاداعي للقلق 4) لا أعرف ماذا أفعل | | | | | |
| --- | --- | --- | --- | --- | --- |
| إذا اخترت الذهاب للاستشارة، أي أخصائي ترغب في استشارته للتقييم التشخيص؟  1) معالج نفسي 2) طبيب الرعاية الصحية للأطفال في المركز الصحي 3) طب اخصائي اطفال 4) طبيب نفسي 5) طبيب جمله عصبيه  6) لااعرف  7) اختصاص طبي اخر | | | | | |
|  | **أوافق بشده** | **أوافق** | **محايد** | **لا اوافق** | **لا اوافق بشده** |
| أشعر **بالخجل** من تشخيص طفلي |  |  |  |  |  |
| جميع الأطفال المصابين بالتوحد عادة ما يعانون من **مشاكل مع العدوانية** |  |  |  |  |  |
| يمكن للأطفال المصابين بالتوحد أن يكبروا **ليعيشوا بشكل مستقل** |  |  |  |  |  |
| التوحد يسبب دائما **إعاقة شديدة** |  |  |  |  |  |
| الأطفال المصابون بالتوحد **سلبيون عمدا وغير ممتثلين** للأوامر |  |  |  |  |  |
| معظم الأطفال المصابين بالتوحد لديهم **مواهب خاصة** |  |  |  |  |  |
| من المهم أن يتلقى الأطفال المصابون بالتوحد خدمات **التعليم الخاص** في المدرسة |  |  |  |  |  |
| يجب **دمج الأطفال** ذوي الاحتياجات الخاصة في المدارس العادية |  |  |  |  |  |
| الأطفال المصابون بالتوحد **أكثر ذكاء** مما تشير إليه الدرجات من الاختبارات |  |  |  |  |  |
| لن يتمكن الأطفال المصابون بالتوحد **من متابعة التعليم** على المستوى الجامعي |  |  |  |  |  |
| **الآباء مسؤولون** عن الحصول على الخدمات لأطفالهم ذوي الاحتياجات الخاصة |  |  |  |  |  |

*These items are used to check the validity of responses, one item should be retained during scoring

**Research questionnaire (scoring)**

**Knowledge**

Regarding the etiology of autism, please select the correct answer

|  | **Yes** | **I don’t know** | **No** |
| --- | --- | --- | --- |
| Autism is caused by **cold-rejecting parents** | 0 | 0 | 1 |
| Autism is caused by **excessively watching TV** | 0 | 0 | 1 |
| **Traumatic experiences** very early in life can cause autism | 0 | 0 | 1 |
| Autism is a **neurodevelopmental disorder** | 1 | 0 | 0 |
| Autism is a **communication disorder** | 0 | 0 | 1 |
| **Unfavorable circumstances** during the first six months of pregnancy | 1 | 0 | 0 |
| Autism is **present only in children** | 0 | 0 | 1 |
| **Genetic factors** play an important role in the development of autism | 1 | 0 | 0 |
| **Vaccines** can cause autism | 0 | 0 | 1 |
| Autism is more commonly diagnosed in **girls** | 0 | 0 | 1 |
| Children who have **siblings with autism** are more likely to develop autism | 1 | 0 | 0 |
| The cause of autism is **not yet known** for sure. | 1 | 0 | 0 |
| Autism is **preventable** | 0 | 0 | 1 |

Regarding symptoms of autism, please select the correct answer**:**

|  | **Yes** | **I don’t know** | **No** |
| --- | --- | --- | --- |
| Autistic children may have **strange reactions** to the way things smell, taste, look, feel, or sound. | 1 | 0 | 0 |
| Many Autistic children have **trouble understanding facial expressions** | 1 | 0 | 0 |
| Some Autistic children **do not talk** | 1 | 0 | 0 |
| Most Autistic children have the **same symptoms** | 0 | 0 | 1 |
| Many Autistic children have **trouble tolerating loud noises or certain types of touch** | 1 | 0 | 0 |
| Many Autistic children show **the need for routines and sameness.** ( get **upset if their routine is changed).** | 1 | 0 | 0 |
| Some Autistic children **show intense interest in parts of objects.** | 1 | 0 | 0 |
| A lot of Autistic children have **problems with**  **being aggressive or hyperactive** | 1 | 0 | 0 |
| Many Autistic children have **difficulty using everyday language** to communicate their needs | 1 | 0 | 0 |
| **Repeating the word** of the others | 1 | 0 | 0 |
| **Delayed response to name** | 1 | 0 | 0 |
| Many times, Autistic children get **excessively focused on one thing** | 1 | 0 | 0 |
| Many Autistic children **repeatedly spin objects or flap their arms.** (having repetitive movements) | 1 | 0 | 0 |
| Most Autistic children may **not look at things when you point at them.** | 1 | 0 | 0 |
| Not being able to look into **the eyes of the person speaking to him** | 1 | 0 | 0 |
| Autistic children **do not enjoy the presence of others** | 1 | 0 | 0 |
| Some Autistic children **may lose acquired speech** | 1 | 0 | 0 |
| **Failure to develop peer relationships** appropriate for developmental age | 1 | 0 | 0 |
| **Social smile is usually absent** | 1 | 0 | 0 |
| **Loss of interest** in their surroundings | 1 | 0 | 0 |
| **Abnormal eating habits** | 1 | 0 | 0 |
| **Lack of spontaneous will to share enjoyment, interest, or activity with others** | 1 | 0 | 0 |
| **Lack of** imaginative play | 1 | 0 | 0 |
| Autism can be **diagnosed as early as 18 months** | 1 | 0 | 0 |

The diagnosis of autism is achieved through (select one)**:**

| **Blood tests** | **Genetic tests** | **Clinical observation** | **Radiological tests** | **I don’t know** |
| --- | --- | --- | --- | --- |
| 0 | 0 | 1 | 0 | 0 |

Treatment of autism includes the following (you can select more than one)**:**

| **Medications** | **Speech therapy** | **Occupational therapy** | **Nutritional therapy** |
| --- | --- | --- | --- |
| 0 | 1 | 1 | 0 |

Regarding the treatment of autism, select the correct answer**:**

|  | **Yes** | **I don’t know** | **No** |
| --- | --- | --- | --- |
| We now have treatments that can **completely** **cure autism** | 0 | 0 | 1 |
| The earlier treatment of autism starts, the **more effective** it tends to be* | 1 | 0 | 0 |
| **Behavior therapy** is an intervention most likely to be effective for Autistic children | 1 | 0 | 0 |
| Autistic children **need extra help to learn** | 1 | 0 | 0 |
| With the proper treatment, most autistic children can eventually **outgrow** autism | 0 | 0 | 1 |
| Earlier treatment demonstrates **no additional benefit to Autistic children*** | 0 | 0 | 1 |
| **Medications** can alleviate core symptoms of autism | 0 | 0 | 1 |

*These items are used to check the validity of responses, one item should be retained during scoring
